# Machine-Learning-Based Muscle Control of a 3D-Printed Bionic Arm

**DOI:** 10.3390/s20113144

**Published:** 2020-06-02

**Authors:** Sherif Said, Ilyes Boulkaibet, Murtaza Sheikh, Abdullah S. Karar, Samer Alkork, Amine Nait-ali

**Affiliations:** 1 College of Engineering and Technology, American University of the Middle East, Al-Eqaila 54200, Kuwait; ilyes.boulkaibet@aum.edu.kw (I.B.); murtaza.sheikh@aum.edu.kw (M.S.); abdullah.karar@aum.edu.kw (A.S.K.); samer.alkork@aum.edu.kw (S.A.); 2 University-Paris-Est, LiSSi, (UPEC), 94400 Vitry-sur-Seine, France; naitali@u-pec.fr

**Keywords:** Myo armband, bionic arm, prosthetic, gesture, recognition, robotics, machine learning

## Abstract

In this paper, a customizable wearable 3D-printed bionic arm is designed, fabricated, and optimized for a right arm amputee. An experimental test has been conducted for the user, where control of the artificial bionic hand is accomplished successfully using surface electromyography (sEMG) signals acquired by a multi-channel wearable armband. The 3D-printed bionic arm was designed for the low cost of 295 USD, and was lightweight at 428 g. To facilitate a generic control of the bionic arm, sEMG data were collected for a set of gestures (fist, spread fingers, wave-in, wave-out) from a wide range of participants. The collected data were processed and features related to the gestures were extracted for the purpose of training a classifier. In this study, several classifiers based on neural networks, support vector machine, and decision trees were constructed, trained, and statistically compared. The support vector machine classifier was found to exhibit an 89.93% success rate. Real-time testing of the bionic arm with the optimum classifier is demonstrated.

## 1. Introduction

Research into advanced medical and prosthetic devices has generated significant attention in recent years due to the increasing demand for reliable bionic hands capable of manifesting the patient’s intentions to perform various tasks. In general, gesture recognition techniques have emerged as a key enabling feature for improving both the accuracy and functionality of bionic hands, allowing the patient control over delicate operations in dangerous situations, or to help patients with movement disorders and disabilities, as well as in the rehabilitation training process.

The use of bionic hands is not only limited to medical use, but has also found innumerable applications in industrial settings; artificial bionic hands can perform certain tasks in hazardous or restricted environments while maintaining the user’s level of dexterity and natural response time. Under such circumstances, vision-based gesture recognition using image detection [1,2,3,4,5,6,7] could be sufficient to provide the correct hand motion.

Furthermore, several technologies, such as electrical impedance tomography (EIT), were used for improving motion detection. Zhang et al. [8] proposed a hand gesture recognition system based on EIT. The EIT system measures the internal electrical impedance and estimates the interior structure by using the surface electrodes and high-frequency alternating current (AC). Although the the proposed system achieves high accuracy, direct contact with the skin is required for proper performance.

Recently, wearable devices based on surface electromyography (sEMG) have become quite attractive in the human gesture recognition domains, as these devices are used to capture the characteristics of the muscles. In general, the sEMG signals obtained from a human arm contain sufficient information with respect to the intended and performed hand gestures [9]. Wheeler et al. [10] introduced a gesture-based control system utilizing sEMG signals taken from a forearm, where the proposed systems were successfully able to act as a joystick movement for virtual devices. Furthermore, Saponas et al. [9] proposed a technique based on ten sEMG sensors worn in a narrow band around the upper forearm to separate the position and pressure of finger presses.

Generally, the feature extraction and pattern recognition stages are very important for the gesture recognition systems to capture gestures well. In the feature extraction stage [11,12], the eigenvalues and the feature vectors for each sEMG sample are selected for classifying the gestures. This procedure can be achieved using several approaches, such as time-domain, frequency-domain, and time–frequency-domain features. On the other hand, the classifier plays an important role in the pattern recognition block, where the most commonly used classifiers are the artificial neural network (ANN), linear discriminant analysis (LDA), and support vector machine (SVM). Li et al. [13] combined force prediction with finger motion recognition, where the time domain and autoregressive methods were both used to extract features. Furthermore, a principal component analysis (PCA) approach was used for further dimensionality reduction, while an artificial neural network classifier was used to evaluate the finger movement. Khezri et al. [14] proposed a system based on the adaptive neuro-fuzzy inference system to recognize six hand gestures. For the feature extractions, the time and frequency domains and their combination were used to extract eigenvectors, and the system provided a recognition rate of 92%. Karlik et al. [15] used the ANN classifier to perform a pattern recognition of six movements of upper limbs. The results were compared with a gesture recognition system based on fuzzy clustering, where the fuzzy clustering neural network has better classification performance than simple ANN algorithms. Similarly, Chu et al. [16] used a wavelet packet transform to extract the feature vectors, and then a dimensional reduction was performed using the PCA algorithm. Finally, a multi-layer ANN is used to classify the gestures. The results show that the eigenvector extraction procedure has more impact on the recognition accuracy than the ability of the classifiers. Huang et al. [17] proposed a system for multilimb movements using the Gaussian mixture model (GMM). The obtained results indicated that the GMM algorithm has a good classification recognition rate at a low computational cost. Noce et al. [18] introduced a new approach for neural control of hand prostheses. This approach is based on pattern recognition applied to the envelope of neural signals. In this approach, sEMG signals were simultaneously recorded from one human amputee, and the envelope of the sEMG signals was computed, while a Support Vector Machine (SVM) algorithm was adopted to decode the user’s intention. The results obtained in this study showed that well-known techniques of sEMG pattern recognition can be used to process the neural signal and can pave the way to the application of neural gesture decoding in upper limb prosthetics. Shi et al. [19] proposed a bionic hand controlled by hand gestures, while the gestures were recognized based on surface EMG signals. The proposed approach was based on extracting multiple features, such as studies—mean absolute value, zero crossing, slope sign change, and waveform length—and a simple k-nearest-neighbors (KNN) algorithm was used as a classifier to perform hand posture recognition. The results show that the KNN classifier was able to recognize four different hand postures.

The key contribution of this work is the design and fabrication of a low-cost 3D-printed bionic arm for an amputee. The proposed solution offers a proof-of-concept demonstration of a 428 g bionic arm with a cost of 295 USD. The Myo arm band technology was leveraged for gesture recognition and training. A comparison between a variety of algorithms based on a neural network, support vector machine, and decision trees was performed to select the most optimal for the proposed design. Furthermore, the best classifier was then used for online gesture recognition and the control of different movements of the bionic hand.

## 2. Bionic Hand

### 2.1. Methodology

**Figure 1:**
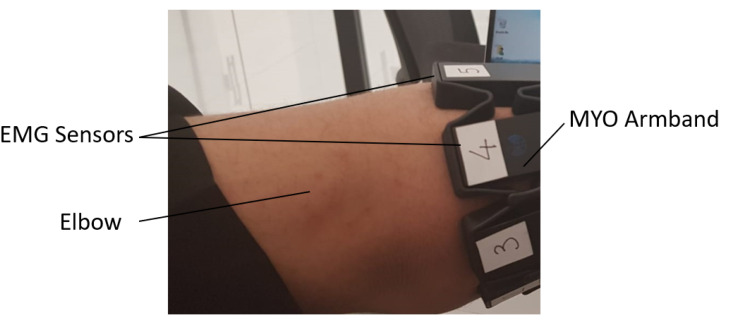
Amputation case with the user wearing a Myo armband.

The bionic arm implemented and tested was customized for a specific user to fit with his amputation conditions. It was made to provide the user with the ability to perform basic grasping actions and allow the user to effectively participate in his daily activities. The user was born with a small portion of his right arm, as shown in the Figure 1. The user is a 24 year old male with no other significant health issues. He used a number of previous prosthetic arms, whose components and make are not detailed. He found that all of these arms are not sufficiently functional or heavy, or are uncomfortable or expensive. The user gave his informed consent for inclusion before he participated in the study. The study was conducted in accordance with the Declaration of Helsinki, and the protocol was approved by the Ethics Committee with reference (SP190402-01).

**Figure 2:**
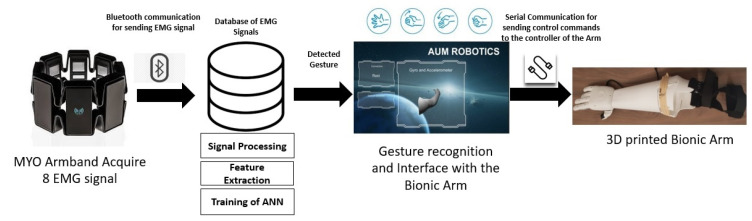
Schematic chart to control the bionic hand.

The user’s feedback was taken into consideration when working on the design of a low-cost customized bionic arm. The user was heavily involved in the mechanical design phase of the bionic arm. The prosthetic arm presented in this work is controlled by a multi-channel sEMG sensor that is used to acquire the muscles’ activities. The muscles’ activities represent different gestures, which are used to perform the required action by the hand attached to the arm. The hand has 9 degrees of freedom (DOF), which enable it to perform different accurate actions as per the user’s demand. The schematic chart illustrating the steps required to control the bionic arm is shown in Figure 2. A database of sEMG gestures is created with all of the units associated with signal processing, feature recognition, and machine learning to catalog the signals that are required for movements of the finger actuators in the bionic hand to perform specific hand postures.

### 2.2. Multi-Channel Wearable Armband

Different wearable sensor systems are available in the market for acquiring different bio-signals, such as electromyography (EMG), electroencephalography (EEG), electrodermal activity (EDA), and electrocardiogram (ECG) [20]. The Myo armband is a wearable armband used primarily for Human Computer Interaction (HCI) applications, where humans can deal with computers and systems in an interactive way. The structure of Myo consists of eight EMG (electromyography) sensors and an inertial measurement unit (IMU), which consists of a gyroscope, accelerometer, and a magnetometer [21]. Due to the engineered design of the multi-channel wearable armband, the user wears it in the small portion of the forearm below the elbow. Myo is used for detecting the different gestures and movements that are applied for sEMG database collection. The database, signal processing, and machine learning techniques described later are used for the accurate detection of four different gestures.

**Figure 3:**
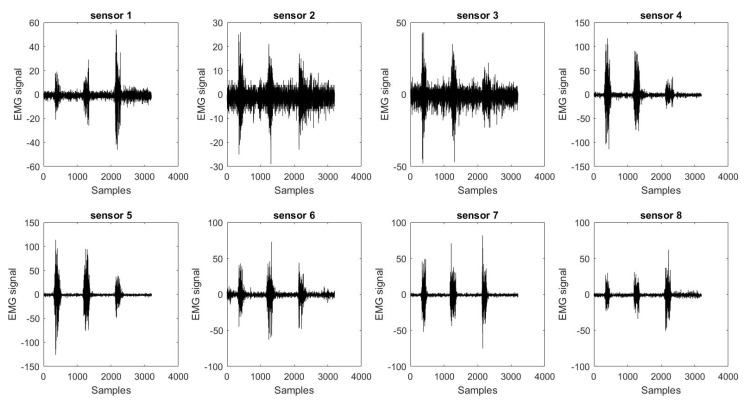
An example of the eight electromyography (EMG) sensors’ raw data collected by the Myo armband.

The user was trained to perform the following four gestures: Fist (close), spread fingers (open), wave-in, and wave-out. The detected gestures were displayed on the screen to provide the user with feedback during the training phase [22]. The concept behind the creation of the sEMG database using Myo is to enable a more generic arm design for any amputee with a similar arm amputation. The combination of the 8 different sEMG electrodes of Myo enabled more sEMG signal data for a better gesture recognition system. Figure 3 shows the raw sEMG signals acquired by Myo while the user is performing hand gestures prior to signal processing. The sEMG sensors of the armband are numbered from 1 to 8 to be able to match the signals with the muscles. Sensor 3, as shown in Figure 1, is placed in the area least affected by the surrounding muscles.

### 2.3. Bionic Arm Design

Amputees with limb amputation may be disappointed with particular aspects of available limbs in the market due to their limitations. A customized design for the user through an individual design process has been undertaken here, which has the capacity to target a design that fulfills the need of an individual amputee case, particularly in terms of its low cost and light weight. The current devices available in the market range from 4000 up to 20,000 USD [23]. A detailed market analysis of the cost associated with prosthetic limbs was compiled by the authors in [24]. A simple cosmetic arm and hand may cost between 3000 and 5000 USD. The cost of a functional prosthetic arm, on the other hand, may cost between 20,000 to 30,000 USD [24]. The main target is to manufacture a cheap and affordable bionic arm for amputees costing around 295 USD. Nowadays, the advancement of and easy access to 3D-printing technology have reduced the cost of manufacturing bionic arms and provide easier solutions for the design of prosthetic arms customized for users [22]. The user’s left arm dimensions were measured to fabricate the right arm with the same dimensions for a symmetric look. A mirrored geometry was assumed using a computer-aided design (CAD) software. The dimensions of the affected arm were taken into consideration and embedded in the design to develop a wearable arm with enough room for the Myo armband to fit and be concealed from view.

**Figure 4:**
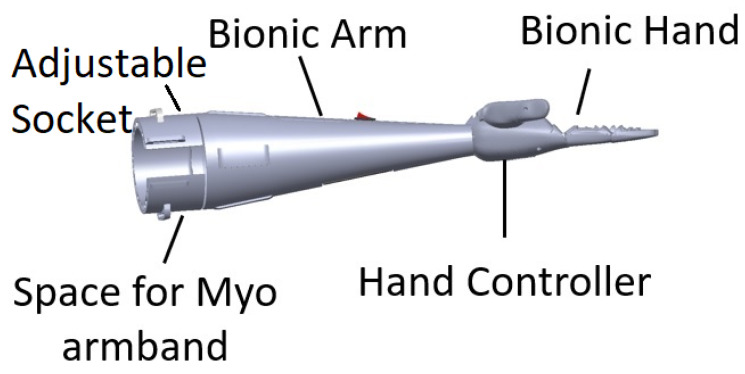
Bionic arm 3D model on computer-aided design (CAD) software.

**Figure 5:**
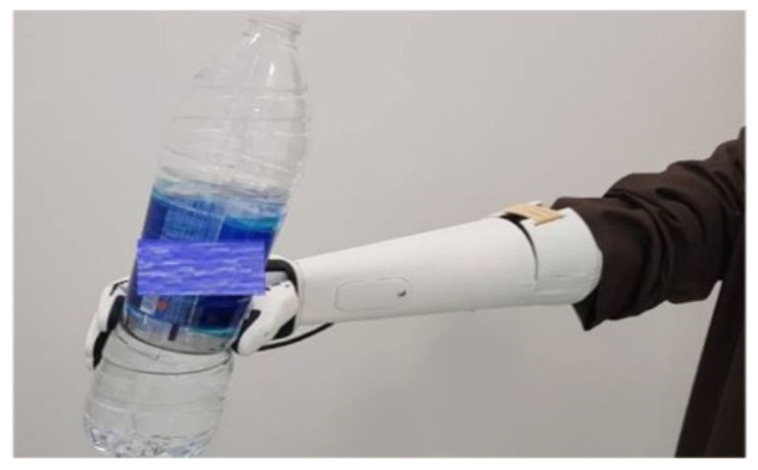
Bionic arm load test.

The 3D model design for the bionic arm is shown in Figure 4. The design is based on different criteria, as listed and described below:

- Adjustable socket: This is the portion that joins the limb (stump) to the bionic arm. The socket designed for this arm is adjusted by a strap. The user is wearing the Myo armband at a set location on his arm before adjusting the size of the socket to have a tight fit. Designs were iteratively created, tested, and the subject’s feedback was taken into consideration, until an improved design was reached, implemented, and tested. The comfort feeling is one of the most important points considered in the design of the socket, allowing the user to mount the bionic arm for up to four hours with the help of the bicep support.
- Dimensions: The symmetry of arm length is critical for the user to avoid serious muscle asymmetry symptoms and muscle pain from disbalance. Consequently, the designed arm was engineered to match the dimensions of the physical left arm.
- Artificial Hand: A 3D model assembled of the open-source Brunel hand was made to ensure the fitting between the arm and the hand. The hand consists of 9 degrees of freedom and 4 degrees of actuation. It is able to perform complex tasks with precision. The four linear motors are attached to threads along with springs to allow smooth linear motion. These linear actuators consist of feedback that allows the control of the location of the fingers precisely.The majority of the parts are printed with polylactic acid (PLA) material to provide a strong structure, whereas the outer layer and the joints are printed with thermoplastic polyurethane (TPU) to provide a soft cushioning and flexible movement. Small printers were used for the small parts and an industrial-size printer for the larger parts. The complete hand fabrication required less than 2 kg of filament. The total weight of the Brunel hand adds up to just below 350 g.
- Bicep Support: An arm harness made of straps was added to release the pressure off the socket joint with a bicep support made of 25 mm width black nylon strap.
- Myo Integration: The Myo armband is integrated in the bionic arm at the position to ensure correct surface electromyography signal capturing.
- Light Weight: The arm is made to be lightweight by strategically designing the arm to fulfill the design requirements ensuring the strength of the bionic arm at the same time. The total weight of the arm, including the hand with the actuators and excluding the Myo armband, is 428 g.
- Stress Analysis: The constructed prototype was tested using finite element analysis software. The software analysis indicates that lifting a 2 kg load is possible with the fixture at the insertion point and the load on the far end, while taking into consideration the 400 g artificial hand. Experimental load tests indicated that the user can carry a maximum load of 4 kg for 10 seconds or 3 kg for 30 seconds before feeling stress on his muscles. A test conducted by the user is to carry a load of 1.5 kg for 60 s, as shown in Figure 5.
- Electronics and Battery: To ensure safe and organized assembly, the electronic wiring and cables were concealed, while the battery was placed in the user’s pocket to minimize weight.

**Figure 6:**
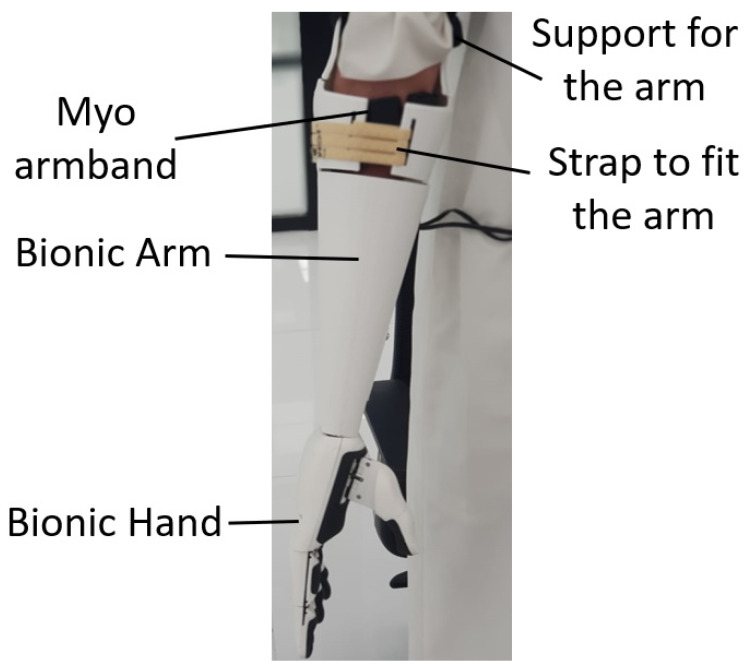
Amputee wearing the bionic arm.

After completion and evaluation of the design, a large-scale industrial 3D printer was used to 3D print the hand parts to be assembled with actuators and electronics. The arm part until the socket was printed in one print. The cost estimation for the arm includes the electronics, actuators, and the 3D-printed material used in the hand. The total cost of the whole arm with parts and electronics is around 295 USD, as detailed in Table 1, which is an affordable price compared to commercially available systems on the market. As the adoption of the proposed arm design will increase the cost of the arm depending on the amputation case, the time for measuring, printing, and assembling is indicated. The final 3D-printed arm is shown in Figure 6.

### 2.4. Electronics and Control

The bionic hand actuators are controlled by a Chestnut board placed inside the bionic hand [25], featuring the ARM Cortex M0+ Processor. The Chestnut board is designed to be embedded within robotic hands. It can control up to four motors simultaneously. The mass of the board is 15 g, and its dimensions are small at 57×45×9 mm, allowing it to fit inside the bionic hand.

**Figure 7:**
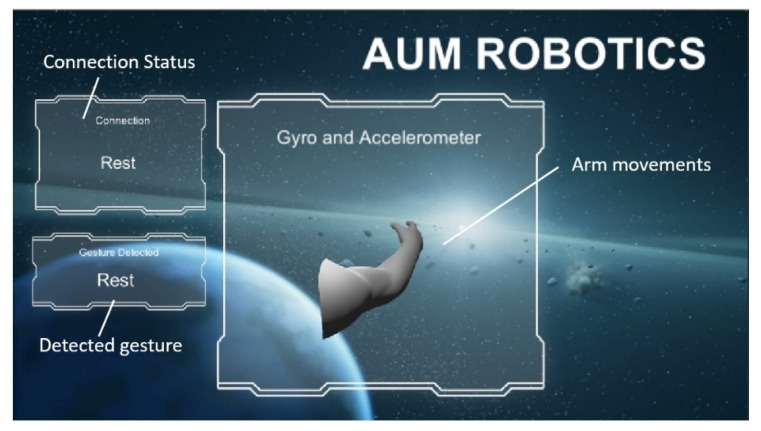
Interface screen on the PC with implemented graphical user interface (GUI).

All of the Myo armband data were acquired wirelessly via Bluetooth at a fixed sampling rate of 200 Hz and transmitted serially to a PC. Each transmitted serial datum corresponds to a gesture. These signals are compared with the trained model of gestures. A graphical user interface (GUI) screen for interfacing with the user was developed to indicate the detected gesture, as shown in Figure 7. The detected gesture by the Myo EMG sensors was mapped to perform hand movements; for example, closing the hand, opening the hand, or closing one finger or two fingers. These actions are achieved by controlling the motion of the actuators. The control signals are transferred through the Chestnut board to move the actuators of the hand.

Although the bionic arm hardware was customized for a single user, the software was meant to be adaptable for any user. Consequently, sets of gesture data were collected from different participants to enable feature extraction and classification, as detailed in the following section.

## 3. Feature Extraction and Classification

In this work, a Myo armband was used to collect the data of the selected four gestures from twenty-three participants (twelve males and eleven females with ages ranging from 18 to 45 years). First, the armband was connected wirelessly to the computer, and several numerical algorithms were used to transform the collected data from the official Myo software, called Myo-Connect, to a matrix data format. This procedure simplified the data collection process and allowed visualization of data while recording. Only data used to train and test the offline classifiers were collected using numerical tools, while the online implementation of this project was be performed using Python code. There are three distinct phases involved: Data collection, data processing and rectification, and feature extraction.

As part of the data collection procedure, participants were instructed to keep an angle of $90^{\circ }$ at the elbow joint during data collection. The dataset was collected in several sessions (within a period of two months), and every time the Myo armband was attached at the same location around the forearm of all participants. In the first phase, data were collected from participants in several sessions, where data associated with four hand gestures were recorded: Spread fingers, closed hand, wave-in, and wave-out. The participants were instructed to move their hand from the resting position to perform one of the proposed gestures and then move back to the resting position for around four seconds. The participants repeated this procedure more than 10 times for each single gesture. The same procedure was applied for all four gestures. As a result, a dataset of 2000 files was collected, where each file contains the signals of several gestures. In the second phase, the collected data were processed and rectified in order to simplify the third phase (the feature extraction phase).

### 3.1. Data Processing

**Figure 8:**
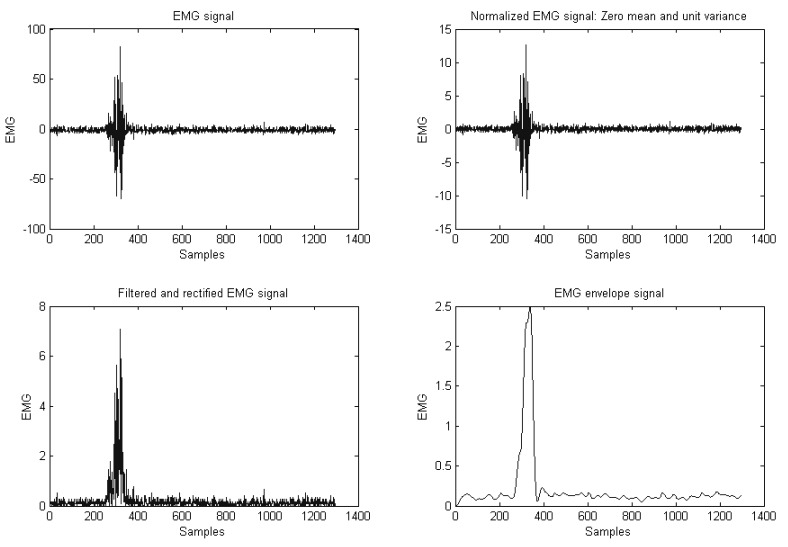
Filtered and rectified EMG signal.

Figure 8 illustrates the second phase of processing raw sEMG signals. First, the raw sEMG signal was modified by removing its mean value, resulting in an AC coupled signal. Next, a band-pass filter was used remove distortions and non-EMG effects from the recorded signal. Generally, raw EMG signals have a frequency between 6–500 Hz. However, certain fast oscillations, which are caused by unwanted electrical noise, may appear within the signal frequency band. Furthermore, slow oscillations, which are caused by movement artefacts or electrical networks, may also contaminate the EMG signals. These unwanted signals can be removed from the original EMG signal using a band-pass filter with cutoff frequencies between 20 and 450 Hz. The resulting data signals may be further rectified by taking the absolute value of all EMG values. This step will ensure that negative and positive values of the EMG signals will not cancel each other upon further analysis, such as calculating the mean values of the absolute EMG signal or obtaining other features. Finally, the second phase was be concluded by capturing the envelope of the filtered and rectified EMG signal, as the obtained shape gives a better reflection of the forces generated by the muscles.

### 3.2. Feature Extraction

In the feature extraction procedure, which is the third phase, the dimensionality of the processed data was reduced in order to simplify the classification step. Generally, sEMG data may contain relevant and irrelevant information, and the irrelevant information can be discarded by mapping sEMG data to another reduced space (reduced dimensionality). This step is known as feature extraction, and the main advantage of this step is the reduction of the dimensionality of the problem, which eventually simplifies the classification process. In this work, a combination of two statistical features, mean absolute value (MAV) and standard deviation (SD), along with the auto-regressive coefficients (AR) approach, is used to extract significant information from the data, which reflects the targeted gestures [26,27,28]. The main idea is to use the sEMG data to fit an auto-regressive model, where the coefficients of the model along with MAV and SD values are then considered as inputs to the classifier for gesture recognition. For each sEMG envelope signal, the AR model is fitted such as:

(1) $$x\left(t\right)-\sum_{k=1}^{m}a_{k}x\left(t-k\right)=e\left(t\right),$$

where $a_{k},k=1,\ldots ,m$, are the AR model parameters, *m* is the order of the model, and e(t) is the error. Then, the parameters $a_{k},k=1,\ldots ,m$ are used to represent the EMG signal. In this work, the value of m=8. A vector of size 2 is needed to capture both MAV and SD values. Furthermore, eight EMG signals were involved in the collection procedure, and the classifier inputs are reduced to eighty entries.

### 3.3. Classification

In this section, the extracted features along with the corresponding known outputs are used as the input data to train a classifier or recognition algorithms. Based on a pre-selected optimization algorithm, the classifier is trained to learn and identify patterns in the data and to respond to the inputs according to the given outputs. After successful training, the reliability of the classifier is tested with a different dataset.

Training and testing classifiers help in validating the results and obtaining an accurate classification model. In this section, three classifiers are investigated: The artificial neural network (ANN), support vector machine (SVM), and decision trees (TD) algorithms, in order to identify which classifier is better suited for building the bionic hand.

#### 3.3.1. Artificial Neural Network

Artificial neural networks (ANN), also known as multi-layer perceptrons (MLP), are one of main pattern recognition techniques; they comprise a large number of neurons, and these neurons are connected in a layered manner. The training procedure of a neural network can be easily achieved by optimizing the unknown weights to minimize a pre-selected fitness function. Generally, the neuron architecture can be summarized as the following: A neuron (or node) receives inputs, and then respective weights are applied on these inputs. Then, a bias term is added on the linear combination of the weighted input signals. The resulting combination is mapped through an activation function. Usually, the ANN consists of input and output layers, as well as hidden layers that permit the neural network to learn more complex features. In this work, one of the most recognized ANN algorithms, the feed-forward neural network, is used as a supervised classifier for gesture recognition. The feed-forward classifier is trained with a set of data (called training data); the trained classifier is then tested with a different dataset. Finally, the resulting ANN classifier is used to recognize online input data [29,30,31,32,33].

#### 3.3.2. Support Vector Machine

A support vector machine (SVM) is a multi-class classifier that has been successfully applied in many disciplines. The SVM algorithm gained its success from its excellent empirical performance in applications with relatively large numbers of features. In this algorithm, the learning task involves selecting the weights and bias values based on given labeled training data. This can be achieved by finding the weights and bias that maximize a quantity known as the margin. Generally, the SVM algorithm was first designed for two-class classification. However, it has been extended to multi-class classification by creating several one-against-all classifiers (in which the algorithm solves *K* two-class problems, and, each time, a class is selected and classified against the rest of the classes), or by formulating the SVM problem as a one-against-one classification problem (in this case, K(K−1)/2 binary classification problems are solved by considering all classes in pairs) [34,35]. In this work, a multi-class SVM classifier is trained, tested, and used to classify gestures based on online data [36].

#### 3.3.3. Decision Tree

Recently, decision tree (DT) algorithms have become very attractive in machine learning applications due to their low computational cost [37]. Furthermore, DT approaches are transparent and easy to understand, since the classification process could be visualized as following a tree-like path until a classification answer is obtained. The decision tree algorithm can be summarized as follows: The classification is broken down into a set of choices, where each choice is about a specific feature. Then, the algorithm starts at the tree’s base (root) and keeps progressing to the leaves in order to receive the optimized classification result. The trees are usually easy to comprehend, and can be transformed into a set of if–then rules, which are suitable for simplifying the training procedure of machine learning applications. Generally, decision trees use greedy heuristic approaches to perform search and optimizations, where these algorithms evaluate their possible options at the current learning stage and select the solution that seems optimal at that instant. In this work, a decision tree algorithm is used to train and test a gesture dataset, and the results are compared with the SVM and ANN in order to select the best model for creating the bionic hand.

## 4. Results

After selecting three different types of classifiers, the offline procedure was used to train and test these classifiers in order to select the model that will be used for the online recognition procedure. The ANN classifier has two hidden layers, with the number of neurons used in each layer set to 116 and 48, respectively. The tanh, which is the hyperbolic tangent function, is considered as the activation function of the ANN. The training procedure is achieved using an optimizer called the limited-memory Broyden–Fletcher–Goldfarb–Shanno (LBFGS) algorithm. In the case of the decision tree classifier, a Gini impurity was used to measure the split’s quality. The lowest number of samples required to split an internal node is considered to be two, and only two samples are required at every leaf node. To obtain an accurate SVM classifier, one should select the right value for the regularization parameter *C*, which is, in this case, C=80, and the kernel parameter γ=0.04.

The parameter values for the three classifiers were selected after performing a cross-validation process for each classifier. Each classifier was used to train and test the same dataset for a different set of parameters. The best model for each version of the three classifiers was selected based on its performance. Next, a statistical study was used to compare the testing results in order to select the best classifier among the three classifiers (ANN, SVM, and DT classifiers). First, each classifier was run for thirty trials, and the testing accuracy for the classification was stored in a table. The SVM classifier provided the highest classification result with a mean value of the training data equal to 91.21% and standard deviation of 1.92%. Furthermore, the SVM classifier provided a testing accuracy equal to 89.93%, and standard deviation of 1.75%. The decision tree algorithm produced a training accuracy of 73.46% with a standard deviation of 4.87%, while the testing results were equal to 70.5% with a standard deviation of 2.5%.

Finally, the ANN classifiers provided a training accuracy of 84.78% with a standard deviation of 4.11%. The ANN accuracy of the testing procedure was equal to 83.91% with a standard deviation of 2.3%. The results are presented in Table 2.

The confusion matrices for the training and testing procedures of the SVM classifier are presented in Table 3 and Table 4, respectively. The four gestures presented in the tables are close, open, wave-in, and wave-out, and the reported results represent a classification trial based on the SVM classifier. As observed, the accuracy for both training and testing procedures was higher than 82%. In addition, the results indicate that the mis-classification between gestures is relatively low and mostly happens between the open and close gestures.

Furthermore, the *t*-test is used to identify if there is a significant difference between the results of all three classifiers. The obtained P-values were found to be quite small (less than 5%), which indicates that there is a significant difference between the classification results. The Holm approach was then used in the statistical investigation to show that there are statically significant differences among the results of the three classifiers, and the SVM classifier provides a better accuracy than both the ANN and the DT classifiers. As a result, the SVM classification model is adopted for the online classification.

## 5. Real-Time Implementation

Different testing protocols were proposed to the user for testing the arm design and the EMG signal control with the optimum classifier enabled. The user practiced for one week on how to perform different gestures and be able to control his muscles. After the training phase, the user wore the Myo armband in his forearm and then performed the trained gestures (fist (closed), spread fingers (open), wave-in, wave-out) using his muscles for 20 consecutive times. Subsequently, the user was asked to perform two different gestures consecutively for 20 times to test the daily activities that can be performed by the bionic arm. The gestures were mapped with four different hand postures, as shown in Table 5.

**Figure 9:**
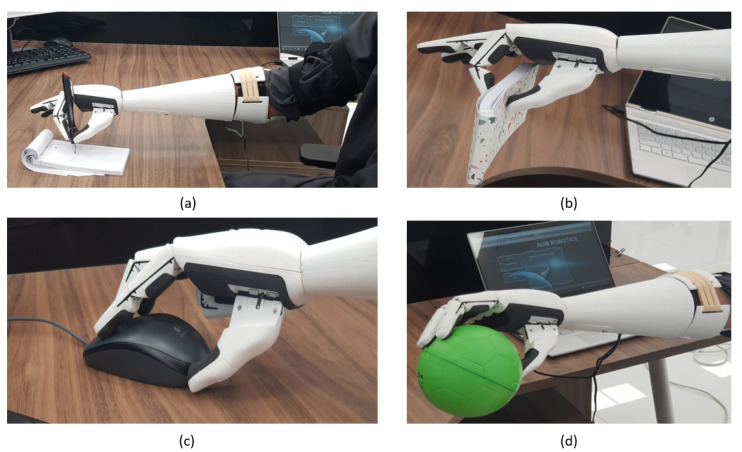
(**a**) Writing with the pen (two fingers closed action); (**b**) holding of a notebook (one finger closed action); (**c**) using the PC mouse (one finger closed action); (**d**) holding a ball (fist action).

The testing scenarios showed the ability of the user to control the bionic hand accurately after the training phase. The bionic hand movements were optimized to allow the user to perform different activities (holding objects, grasping, drinking, and writing). In single-action testing, the user was asked to perform one action at a time. The single actions include making a fist, spreading the fingers, closing one finger, and closing two fingers, as shown in Figure 9.

**Figure 10:**
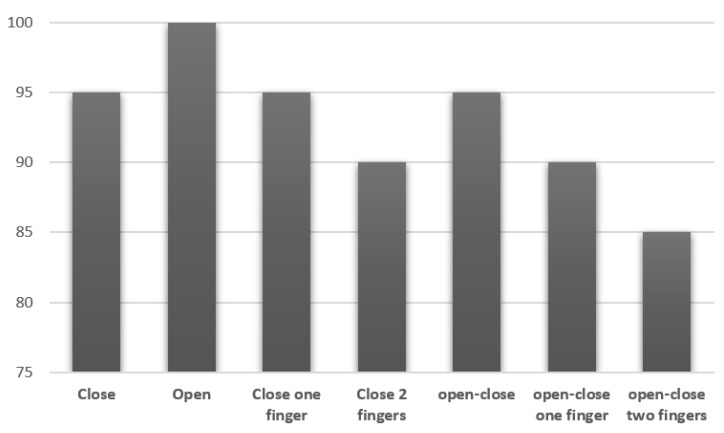
Success rate of hand actions.

The user performed each action repetitively for 20 consecutive times. The results of testing each single action show a minimum detection rate of 85%. In the combination of two actions, the user performed opening and closing with a success rate of 95%, opening and closing of one finger with 90%, and opening and closing of two fingers with 85%, as shown in Figure 10.

## 6. Conclusions and Recommendations for Future Work

A customized 3D-printed bionic arm was designed, fabricated, and tested for a right arm amputee. The 3D-printed bionic arm was designed to have a low cost, comfort, light weight, durability, and appearance. To facilitate a generic control of the bionic arm, EMG data were collected for a set of four gestures (fist, spread fingers, wave-in, wave-out) from a wide range of participants. The collected data were processed and feature extraction was performed for the purpose of training a classifier. The support vector machine classifier was found to out-perform the neural network and decision tree classifiers, reaching an average accuracy of 89.93%. Real-time testing of the bionic arm with the associated classifier software enabled the user to perform his daily activities.

Additional features are needed to further improve the bionic arm; for example, a multi-degree-of-freedom wrist joint connector. This can be achieved by using two servo motors with brackets or by utilizing a spherical manipulator. Furthermore, air-ducted adjustable sockets can allow the user to mount and dismount the bionic arm with ease.

## Figures and Tables

**Table 1:** Detailed cost analysis of the bionic arm.

Index	Property	Value
1	Time to print and assemble the hand	28 h
2	Time to print the arm	10 h
3	Total weight without support material	78.78 g
4	Material cost	$12.41
5	Material cost	$20
5	Hand print	$20
6	Electronics	$20
7	Actuators	$240
	Total cost	$295

**Table 2:** Training and testing results for the three classifiers.

Method	Training	Testing
**SVM**	91.21% ± 1.92%	89.93% ± 1.75%
**ANN**	84.78% ± 4.11%	83.91% ± 2.30%
**DT**	73.46% ± 4.87%	70.51% ± 2.51%

**Table 3:** Confusion matrix for the support vector machine (SVM) classifier: Training (accuracy: 93.75%).

	Close	Open	W-in	W-out
**Close**	91.23%	5.26%	0%	3.51%
**Open**	3.34%	95%	0%	1.66%
**Wave-in**	0%	3.64%	96.36%	0%
**Wave-out**	4.41%	0%	2.94%	92.65%

**Table 4:** Confusion matrix for the SVM classifier: Testing (accuracy: 92.62%).

	Close	Open	W-in	W-out
**Close**	94.64%	0%	3.57%	1.79%
**Open**	6.35%	88.89%	0%	4.76%
**Wave-in**	3.75%	0%	96.30%	0%
**Wave-out**	8.45%	0%	0%	91.55%

**Table 5:** Mapping between the trained gestures and hand actions.

Gesture	Hand Action
Close	Closed hand and fingers
Open	Open hand and fingers
Wave-in	Closing one finger
Wave-out	Closing two fingers
